# Beyond Traditional Use of *Alchemilla vulgaris*: Genoprotective and Antitumor Activity In Vitro

**DOI:** 10.3390/molecules27238113

**Published:** 2022-11-22

**Authors:** Sanja Jelača, Zora Dajić-Stevanović, Nenad Vuković, Stefan Kolašinac, Antoaneta Trendafilova, Paraskev Nedialkov, Miroslava Stanković, Nasta Tanić, Nikola T. Tanić, Aleksandar Acović, Sanja Mijatović, Danijela Maksimović-Ivanić

**Affiliations:** 1 Department of Immunology, Institute for Biological Research “Siniša Stanković”—National Institute of the Republic of Serbia, University of Belgrade, Bulevar Despota Stefana 142, 11060 Belgrade, Serbia; 2 Faculty of Agriculture, University of Belgrade, Nemanjina 6, 11080 Belgrade, Serbia; 3 Department of Chemistry, Faculty of Science, University of Kragujevac, Radoja Domanovića 12, 34000 Kragujevac, Serbia; 4 Institute of Organic Chemistry with Centre of Phytochemistry, Bulgarian Academy of Sciences, Acad. G. Bonchev Str., bl. 9, 1113 Sofia, Bulgaria; 5 Department of Pharmacognosy, Faculty of Pharmacy, Medical University of Sofia, Dunav Str. 2, 1000 Sofia, Bulgaria; 6 Laboratory for Radiobiology and Molecular Genetics, Institute of Nuclear Sciences—National Institute of the Republic of Serbia, University of Belgrade, Mike Petrovića Alasa 12–14, 11351 Belgrade, Serbia; 7 Department of Dentistry, Faculty of Medical Sciences, University of Kragujevac, Svetozara Markovića 69, 34000 Kragujevac, Serbia

**Keywords:** *Alchemilla vulgaris* L., antitumor action, antioxidative activity, genoprotective effect

## Abstract

*Alchemilla vulgaris* L. (lady’s mantle) was used for centuries in Europe and Balkan countries for treatments of numerous conditions and diseases of the reproductive system, yet some of the biological activities of lady’s mantle have been poorly studied and neglected. The present study aimed to estimate the potential of *A. vulgaris* ethanolic extract from Southeast Serbia to prevent and suppress tumor development in vitro, validated by antioxidant, genoprotective, and cytotoxic properties. A total of 45 compounds were detected by UHPLC–HRMS analysis in *A. vulgaris* ethanolic extract. Measurement of antioxidant activity revealed the significant potential of the tested extract to scavenge free radicals. In addition, the analysis of micronuclei showed an in vitro protective effect on chromosome aberrations in peripheral human lymphocytes. *A. vulgaris* extract strongly suppressed the growth of human cell lines derived from different types of tumors (MCF-7, A375, A549, and HCT116). The observed antitumor effect is realized through the blockade of cell division, caspase-dependent apoptosis, and autophagic cell death. Our study has shown that *Alchemilla vulgaris* L. is a valuable source of bioactive compounds able to protect the subcellular structure from damage, thus preventing tumorigenesis as well as suppressing tumor cell growth.

## 1. Introduction

Ethnopharmacological data are of crucial importance for the finding of new promising bioactive compounds, as well as for the verification of already accepted herbal drugs. Therefore, it is necessary to preserve the traditional knowledge of medicinal plants in addition to addressing the need for their sustainable collection from the wild. Nowadays, therapeutic approaches have switched from attacking and directly destroying the damaged cells and pathogenic microorganisms towards the activation of self-healing and protective processes upon the initiation of different repair mechanisms of the human body. Such a view has a great impact on scientific research focusing on the bioactivity of natural products [1]. Numerous pathological conditions cannot be fully treated by standard pharmaceutics [2], and thus some plant drugs and extracts are considered a good alternative to conventional drugs due to their synergistic properties and minimal side effects [3].

Among plants with a long history in folk medicine and consequent recognition in different pharmacopeias are the species of the genus *Alchemilla*.

The genus *Alchemilla* L. comprises over 300 species of clump-forming, herbaceous perennials growing on upland wet meadows in Europe, Western Asia, and North America, but they are also found in mountain regions of South America and Africa [4,5]. *Alchemilla vulgaris* L., commonly known as lady’s mantle, is the most studied species of the genus. The recent taxonomic interpretations of *A. vulgaris* assumed that the taxon is an aggregate comprising 12 apomictic morphologically similar microspecies which frequently hybridize [6]. The European Pharmacopoeia referred to *Alchemilla vulgaris* L. *sensu latiore* [4].

Lady’s mantle is widely used in folk medicine throughout the world. The upper parts of the plant were reported for treating diabetes, multiple sclerosis, anemia, ulcers, hernias, gynecological and abdominal disorders, wounds, rashes, and inflammations [7,8]. In Southeast Europe and the Balkans, *Alchemilla* species are used for gynecological, menstrual, and menopausal complaints; respiratory infections; diarrhea; diabetes; kidney and liver diseases; weight loss; skin disorders; and different inflammatory conditions [9,10,11].

In addition, this medicinal plant exhibits antibacterial, antifungal, and antiviral properties [10,11]. In the context of bacterial resistance to antibiotics, plant drugs and extracts are considered potent antibacterial agents without the risk of a further increase in resistance to standard antimicrobial agents [12]. A recent study targeting the effects of the lady’s mantle infusion after hypoxic exposure indicated the neuroprotective properties of the plant drug [13]. Neagu et al. demonstrated the acetylcholinesterase and tyrosinase inhibitory effect of *Alchemilla vulgaris* extract, and therefore it was assumed that the species could be used in the prevention and treatment of neurodegenerative diseases [14]. So far, some of the biological activities of lady’s mantle have been poorly studied and neglected. Namely, the anticancer activity of *Alchemilla vulgaris* has been reported previously by Vlaisavljević et al. and Ibrahim et al. While Vlaisavljević et al. reported strong anticancer activity of *Alchemilla vulgaris* against estrogen-dependent tumors of female reproductive organs, Ibrahim et al. demonstrated cytotoxic activity of *Alchemilla vulgaris* root methanolic extract against several other cell lines in vitro [15,16]. Apart from genoprotective and antioxidant activity, this study has shown for the first time the potential of an ethanolic extract of aerial parts of *Alchemilla vulgaris* L. to decrease the malignant potential of hormone-independent tumor cell lines through the blockade of cell division, as well as induction of programmed cell death types I and II.

## 2. Results and Discussion

### 2.1. Phytochemical Characterization of A. vulgaris Ethanolic Extract—UHPLC−HRMS

The ethanolic extract of *A. vulgaris* was investigated by UHPLC-HRMS. A total of 45 compounds (Table 1) were tentatively characterized based on their chromatographic behavior parameters such as retention time, *m*/*z* values, molecular formula, error, and fragmentation pattern and comparison with those described in the literature and open access LC-MS libraries. The identified compounds belong to different metabolic classes, mainly phenolics.

#### 2.1.1. Flavonol and Flavone Glycosides

Twenty-two flavonol derivatives and two flavone glycosides were detected in *A. vulgaris* extract and represented the main group of metabolites. The majority of these compounds were quercetin and kaempferol derivatives identified based on their abundant fragment ions appearing at *m*/*z* 301 for quercetin (**8**, **9**, **20**, **21**, **24**–**26**, **29**, **31**, **32**, and **36**) and at *m*/*z* 285 for kaempferol (**28**, **33**–**35**, **39**, and **41**).

Quercetin (**40**) was identified by the deprotonated molecular ion at *m*/*z* 301 [M − H]^−^ and prominent fragments at *m*/*z* 179.00 and *m*/*z* 151.00 obtained by RDA fragmentation in the MS/MS spectrum [28]. The neutral loss of 176 (compound **26**), 162 (**25** and **29**), and 132 Da (**31** and **32**) from the precursor ion in MS/MS spectra revealed the presence of glucuronic acid, hexose, and pentose moieties. Further, the higher intensity of the quercetin radical anion [Y_0_ − H]^−^ at *m*/*z* 300 in comparison with that at *m*/*z* 301 [Y_0_]^−^ along with the characteristic fragments at *m*/*z* 271 [Y_0_ − H − CO − H]^−^ and 255 [Y_0_ − H − CO_2_ − H]^−^ determined these compounds as quercetin 3-*O*-monoglycosides [18,21]. Thus, compound **26** was identified as quercetin-3-*O*-glucuronide (miquelianin), **25** and **29** as quercetin-3-*O*-galactoside (hyperoside) and quercetin-3-*O*-glucoside (isoquercitrin), and the pair 31/32 as quercetin 3-*O*-α-L-arabinopyranoside (guaijaverin) and quercetin 3-*O*-α-L-arabinofuranoside (avicularin). Compound **24** showed [M − H]^−^ at *m*/*z* 609 and a fragmentation pattern similar to that described above. Therefore, 24 was identified as rutin (quercetin 3-*O*-rutinoside). Compounds **9** and **20** were quercetin diglycosides as they displayed the same [M − H]^−^ ion at *m*/*z* 595 but differed in the fragmentation pattern of the precursor ion. Thus, the MS/MS spectrum of the precursor ion of compound **9** showed [M − H−132]^−^ and [M − H − 133]^−^ (*m*/*z* 463 and 462) and [M − H − 162]^−^ (*m*/*z* 433) due to the loss of pentose and hexose moieties as well as [Y_0_ − 2H]^−^ (*m*/*z* 299) characteristic of quercetin 3,7-*O*-diglycosides. Compound **9** was identified as quercetin-3-*O*-arabinoside-7-*O*-glucoside based on the higher intensity of the peak at *m*/*z* 433 in comparison with that at *m*/*z* 463 [21]. Further, the base peak at *m*/*z* 300 [Y_0_ − H]^−^ and prominent peaks at *m*/*z* 271 and 255 determined compound **20** as quercetin-3-*O*-vicianoside (quercetin 3-*O*-α-L-arabinopyranosyl-(1–6)-β-D-glucopyranoside). Compound **21** showed [M − H]^−^ at *m*/*z* 579. Its MS/MS spectrum showed two fragment ions at *m*/*z* 447 and 433 due to the loss of a pentose unit (132 Da) and a deoxyhexose unit (146 Da) and fragment ions [Y_0_ − 2H]^−^ (*m*/*z* 299) and [Y_0_ − H − CO − H]^−^ (*m*/*z* 271), characteristic of a quercetin aglycone. The higher intensity of the peak at *m*/*z* 447 in comparison with that at *m*/*z* 433 supported the attachment of the pentose and deoxyhexose moieties at C-3 and C-7 of the quercetin molecule, respectively. Therefore, compound **21** was identified as quercetin-3-*O*-pentoside-7-*O*-deoxyhexoside. Compound **8** was tentatively identified as quercetin hexoside-glucuronide due to the presence of a deprotonated molecular ion at *m*/*z* 639 and prominent peaks *m*/*z* 301 [M − H − 338]^−^ (loss of hexoside-glucuronic unit) and 463 [M − H − 176]^−^ (loss of glucuronide unit). Compound **36** was identified as quercetin 3-*O*-acetylglucoside due to the presence of a molecular ion [M − H]^−^ at *m*/*z* 505 and a product ion at *m*/*z* 300 (loss of an acetylglucosyl unit, 205 Da). Quercetin (**40**) and its glycosides (**9**, **24**–**26**, **29**, and **32**) have been described as components of *A. vulgaris* [11,15,19]. Guaijaverin (**31**), diglycosides (**20**, **21**), and quercetin 3-*O*-acetylglucoside (**36**) are described for the first time as components of *A. vulgaris*. Guaijaverin (**31**) was isolated previously from *A. xanthochlora* Rothm. [30], *A. achtarowii* [31], and *A. jumrukczalica* [32].

Compounds **28**, **33**–**35**, **39**, and **41** were kaempferol 3-*O*-glycosyl derivatives as their MS/MS spectra exhibited characteristic ions at *m*/*z* 284 [Y_0_ − H]^−^, 285 [Y_0_]^−^, 255 [Y_0_ − H − CO − H]^−^, and 227. Further, the loss of 308 Da compound **28**, 176 Da compound **33**, 162 Da compound **34**, and 132 Da compound **35** from the deprotonated molecular ions revealed the presence of rutinose, glucuronic acid, hexose, and pentose moieties [18,21]. Thus, compounds were identified as kaempferol-3-*O*-rutinoside (**28**), kaempferol-3-*O*-glucuronide (**33**), kaempferol-3-*O*-glucoside astragalin, (**34**), and kaempferol-3-*O*-xyloside (**35**). Compounds **39** and **41** were identified as kaempferol 3-*O*-acetylglucoside and kaempferol 3-*O*-(6′′-*O*-p-coumaroyl)-β-D-glucopyranoside (tiliroside) as their spectra showed [M − H]^−^ at *m*/*z* 489 and 593, corresponding to molecular formula C_23_H_21_O_12_ and C_30_H_25_O_13_ and a fragment ion at 285 due to the elimination of acetylglucose (−205 Da) and coumaroylglucose (−308 Da) units, respectively [27]. All kaempferol compounds with exception of kaempferol-3-*O*-glucoside (**34**) and tiliroside (**41**) are described now for the first time as components of *A. vulgaris*. Kaempferol-3-*O*-glucuronide (**33**) has been previously isolated from *A. speciosa* [33].

Compounds **12** and **17**–**19** were identified as myricetin and gossypetin glycosides, respectively, by comparing their retention times and mass spectral behavior with those published in the literature [28]. Myricetin and gossypetin are pentahydroxyflavonols differing in the position of one OH group and gave similar fragmentation in their MS/MS spectra with ions [Y_0_]^−^ and [Y_0_ − H]^−^ (*m*/*z* 317 and 316) and [Y_0_ − H]^−^ and [Y_0_ − H − CO − H_2_O]^−^ (*m*/*z* 271). Compound **12** was identified as myricetin 3-*O*-hexoside as it displayed [M − H]^−^ at *m*/*z* 479 and an MS/MS fragment due to the loss of hexose (162 Da) at *m*/*z* 317 [Y_0_]^−^ and the higher intensity of the peak at *m*/*z* 316 [Y_0_ − H]^−^ [21]. Compounds **17**–**19** were gossypetin diglycosides as they exhibited [M − H]^−^ at *m*/*z* 625 (**17**) and 595 (**18** and **19**) and fragments in MS/MS spectra formed by elimination of two individual sugar units: pentose and hexose in (**17**) (*m*/*z* 479 [M − H − 146]^−^ and 463 [M − H − 162]^−^) and pentose and deoxyhexose in (**18** and **19**) (*m*/*z* 463 and 449). The higher intensity of the peak at *m*/*z* 479 in comparison with those at *m*/*z* 463 (in **17**) and 449 (in **18** and **19**) supported the attachment of the pentose and hexose/deoxyhexose moieties at C-3 and C-7 of the gossypetin molecule, respectively. The compounds **17**–**19** were tentatively identified as gossypetin 3-*O*-hexoside-7-*O*-rhamnoside and gossypetin 3-*O*-deoxyhexoside-7-*O*-rhamnoside isomers 1 and 2 [21,24,28]. All these compounds are described for the first time in *A. vulgaris*. However, the presence of gossypetin glycosides in the studied extract was not very surprising as gossypetin derivatives were isolated from *A. mollis* [34]. It is worth mentioning that myricetin derivatives have not been detected in *Alchemilla* species so far.

Compound **30** showed [M − H]^−^ at *m*/*z* 447. The MS/MS spectrum presented the characteristic fragmentation patterns for luteolin (*m*/*z* 285 and 199) [22]. Therefore, compound **30** was identified as luteolin 7-*O*-glucoside. This compound has been already described as a component of *A. vulgaris* [11].

Compound **38** showed [M − H]^−^ at *m*/*z* 475 releasing MS/MS fragments at *m*/*z* 299 [M − H − 176]^−^ and 284 [M − H − 176 − 15]^−^ attributed to the successive loss of a glucuronyl moiety and a methyl group and was identified as chrysoeriol 7-*O*-glucuronide [26]. Compound **38** is reported now for the first time in *A. vulgaris*, although chrysoeriol has been previously detected in *A. vulgaris* [15].

#### 2.1.2. Phenolic Acids and Their Derivatives

Gallic acid (**1**), chlorogenic acid (**3**), caffeic acid (**7**), syringic acid (**11**), *p*-coumaric acid (**13**), and *p*-coumaroylquinic acids (**10** and **15**) were identified in the studied *A. vulgaris* extract. The spectra generated for these compounds in negative ion mode gave the deprotonated molecule [M − H]^−^ and a characteristic product ion [M − H − 44]^−^ due to the loss of CO_2_ in their MS/MS spectra [17,18,35]. The MS/MS spectrum of chlorogenic acid (**3**) contained also characteristic product ions at *m*/*z* 191, 179, and 173 corresponding to the loss of quinic acid [M − H − 162]^−^, a caffeic acid unit, and the loss of CO_2_ [191 − 44]^−^. The MS/MS spectra of compounds **10** and **15** showed the typical fragmentation of the *p*-coumaric acid at *m*/*z* 163 and 119 and the loss of the *p*-coumaric acid unit at *m*/*z* 191 [18,35].

The spectra of compounds **2** and **6** showed ions *m*/*z* 325 (C_15_H_17_O_8_) and *m*/*z* 163 corresponding to the deprotonated molecule [M − H]^−^ and the loss of a glucose unit at *m*/*z* 163 and were identified as *p*-coumaroyl hexose isomers [18,36,37].

Ellagic acid (**23**) had [M − H]^−^ at *m*/*z* 301 and characteristic fragment ions at *m*/*z* 283 [M − H − H_2_O]^−^, 229 [M − H − CO_2_ − CO]^−^, 201 [M-H − CO_2_ − CO − CO]^−^, and 185 [M − H − 2CO_2_ − CO]^−^, formed from the precursor ion in MS/MS spectrum [19,23].

Compounds **16** and **22** had the deprotonated molecules [M − H]^−^ at *m*/*z* 463 and 433 and the product ion at *m*/*z* 301 in their MS/MS spectra due to the loss of a hexose (−162 Da) and pentose (−142 Da) as well as a typical fragmentation pattern for ellagic acid. Therefore, these compounds were tentatively identified as ellagic acid-hexose and ellagic acid-pentose [23].

A literature survey showed that gallic acid (**1**), chlorogenic acid (**3**), caffeic acid (**7**), *p*-coumaric acid (**13**), and ellagic acid (**23**) were detected in *A. vulgaris* extracts [11,15,19], while all others are found for the first time in *Alchemilla* species.

#### 2.1.3. Ellagitannins and Other Phenolic Compounds

Compound **4** had [M − H]^−^ at *m*/*z* 633 and major fragment ions at *m*/*z* 463 [M − H − 170]^−^ and 301 [M − H − 170 − 162]^−^ due to a sequential loss of gallic acid and one hexose group, bonded to a hexahydroxydiphenoyl group (HHDP) unit. Therefore, compound **4** was identified as galloyl-HDDP-hexose, previously found in *A. vulgaris* and *A. mollis* [19].

Compound **27**, agrimoniin, showed a fragment at *m*/*z* 934 [M − 2H]^2−^, corresponding to one galloyl-bis-glucose unit, followed then by fragmentation ions at *m*/*z* 633 and 301 due to the loss of an HHDP unit (302 Da) and a galloylglucose residue (332 Da) [25]. Agrimoniin is a common ellagitannin in *Alchemilla* species [19,25].

Compounds **14** and **5** showed identical fragmentation patterns. Compound **14** with a molecular formula of C_12_H_8_O_6_ and a protonated molecule [M − H]^−^ at *m*/*z* 247 generated product ions at *m*/*z* 219 and 191, resulting from the successive loss of a CO unit. Similarly, in the case of compound **5**, the major product ion at *m*/*z* 247 was formed via decarboxylation of its precursor ion at *m*/*z* 291. The further fragmentation was consistent with that mentioned above for compound **14**. According to the literature data, compounds **14** and **5** were identified as brevifolin and brevifolincarboxylic acid [20,25]. Moreover, brevifolincarboxylic acid has been recently described as a component of *A. viridiflora* [25].

#### 2.1.4. Triterpenoids

Compounds **37** and **42**–**44** were tentatively identified as triterpene acid hexosides by comparison of their mass spectral characteristics with those previously reported [25]. Their precursor ions at *m*/*z* 711 (**37**) and 695 (**42**–**44**) and the major fragments at *m*/*z* 503 and 487 [M + HCOO − 162]^−^, respectively, were in accordance with the formate adducts of hexosyl esters of tetrahydroxy (**37**) and trihydroxy (**42**–**44**) pentacyclic triterpene acids of ursane and/or oleane type. Compound **45** was tentatively identified as arjungenin (2,19,23-trihydroxyoleanolic acid) as it showed [M − H]^−^ at *m*/*z* 503 and a characteristic fragment at *m*/*z* 409 [29]. This is the first report of triterpene acid glycosides in *A. vulgaris*. A literature survey revealed the presence of ursolic, oleanolic, 2a-hydroxyursolic, 2,19-dihydroxyursolic (tormentic acid), and 2,3,19-trihydroxyurs-12-en-28-oic acid (euscophic acid) acids in *A. vulgaris*, *A. faeroensis*, and *A. alpina* [38].

### 2.2. Total Phenolics and Flavonoids and Antioxidant Activity

The results presented in Table 2 represent the results of total phenolic and flavonoid contents and antioxidant capacity. Results for TPC are in accordance with results obtained by Vlaisavljevic et al. [15]. According to Boroja et al., results for TAC and DPPH were lower than those in the current study, which might be a consequence of different solvents, extraction procedures, and the used standard chemicals in performed assays [39]. However, our results are in line with other studies on the antioxidant capacity of the *Alchemilla* extracts [7,34,40], all indicating the strong correlation between high phenolic content and antioxidant activity, especially concerning a high share of the total flavonoids and tannins in the plant extract [15,41].

### 2.3. Genoprotective Effect of A. vulgaris Extract

Three different concentrations of *A. vulgaris* extract were tested in vitro for protective effect on chromosome aberrations in peripheral human lymphocytes using a CBMN assay: 2.0 µg/mL, 4.0 µg/mL, and 6.0 µg/mL. The frequency and distribution of MN were scored. The formation of MN after treatment with an alkylating agent, mitomycin C (MMC), and the prevention of MN formation after treatment with DNA repair system agent amifostine WR-2721 were determined. The test system was a peripheral human blood lymphocyte assay, verifying the clastogenic or anticlastogenic effects [42,43]. The possible clastogenic, anticlastogenic, or modulating effects of investigated compounds were determined based on the action of these two agents. The results are presented in Table 3.

**Figure 1:**
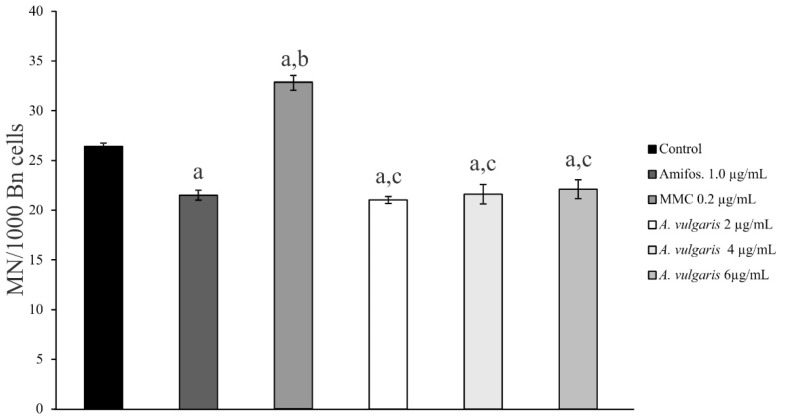
Incidence of MN measurement in cell cultures of human lymphocytes treated with different concentrations of *A. vulgaris* extract. The statistical significance of the difference between the data pairs was evaluated by analysis of variance (one-way ANOVA) followed by the Tukey test. Statistical difference was considered significant at *p* < 0.01. a: Compared with control groups, statistically significant difference *p* < 0.01. b: Compared with amifostine—WR 2721, statistically significant difference *p* < 0.01. c: Compared with mitomycin C, statistically significant difference *p* < 0.01.

Lymphocyte cell culture treated with 1 μg/mL of amifostine WR-2721 showed a significant decrease (*p* < 0.01) of 18.6% in the frequency of MN compared to control cell cultures (Table 3, Figure 1). Treatment with an MMC alkylating agent at the concentration of 0.2 µg/mL showed a significant increase (*p* < 0.01) in MN frequency of 24.2% compared to control cell cultures (Table 3, Figure 1).

Three different concentrations of *A. vulgaris* extract were tested for in vitro protective effect on chromosome aberrations in peripheral human lymphocytes using cytochalasin-B-blocked MN assay. *A. vulgaris* extract at concentrations of 2.0 µg/mL, 4.0 µg/mL, and 6.0 µg/mL caused a slight decrease in the MN frequency by 20.5%, 18.2%, and 16.3%, respectively, when compared to the control cell cultures (Table 3, Figure 1). Most importantly, *A. vulgaris* extract at the concentration of 2.0 µg/mL still had a higher protective effect than the synthetic protector, amifostine WR-2721, at the concentration of 1.0 µg/mL (Table 3, Figure 1).

The effect of different concentrations of *A. vulgaris* extract on cell proliferation was investigated by determination of the cytokinesis-block proliferation index (CBPI). Table 3 shows mean CBPI values and standard errors calculated for different concentrations of *A. vulgaris* extract. The comparable CBPI values of extracts and amifostine WR-2721 control suggest an inhibitory effect of the tested extracts on lymphocyte proliferation. In this study, we found that the lower concentrations of *A. vulgaris* extract possess a beneficial effect on lymphocyte cell culture by decreasing the frequency of MN. Since the number of micronuclei serves as an indicator of DNA damage, these results indicate that *A. vulgaris* extract protects DNA and decreases lipid peroxidation of lymphocytes mostly induced by superoxide anion radicals. The free radicals disturb cellular homeostasis by peroxidation of membrane lipids, oxidation of proteins, base damage, and adduct formation in DNA, which ultimately leads to cell death if the damage is beyond cell repair capacity [44,45,46].

### 2.4. Antitumor Property of A. vulgaris Extract

**Figure 2:**
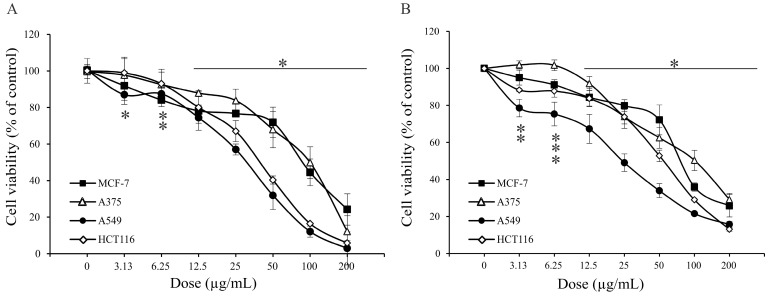
*A. vulgaris* extract suppresses the growth of all tumor cell lines. After 72 h of treatment, the cell viability of MCF-7, A375, A549, and HCT116 cells was estimated by (**A**) MTT assay and (**B**) SRB assay. * indicates statistically significant (*p* < 0.05) values in comparison to the control. The two or three vertical * correspond to the separated values belonging to different curves which are close or overlapping.

**Figure 3:**
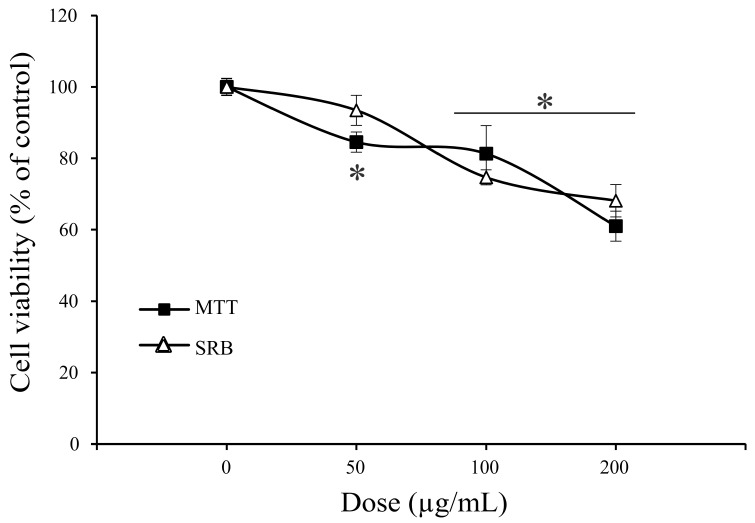
*A. vulgaris* extract showed no inhibitory effect on primary cells. Viability of primary peritoneal exudate cells by MTT and SRB tests upon treatment with *A. vulgaris* extract for 72 h. * indicates statistically significant (*p* < 0.05) values in comparison to the control.

Human hormone-dependent breast cancer MCF-7, anaplastic melanoma A375, lung adenocarcinoma A549, and colon carcinoma HCT116 cell lines were exposed to a wide range of concentrations of *A. vulgaris* ethanolic extract, and after 72 h of incubation, cell viability was determined by the measurement of mitochondrial respiration or protein synthesis, using MTT and SRB tests, respectively. As shown in Figure 2, data obtained in both assays confirmed a strong dose-dependent viability decrease in all tested cultures exposed to *A. vulgaris* extract, apart from malignant cells’ origin and characteristics. Since the viability of primary peritoneal exudate cells isolated from healthy animals was not affected by the same range of doses under a comparable experimental setting (Figure 3), it can be concluded that *A. vulgaris* extract displayed selectivity for the malignant phenotype that is even independent of the hormonal status as believed previously [15]. IC_50_ values of all cell lines (Table 4) illustrated the highest effectiveness of *A. vulgaris* extract against hormone-independent A549 and HCT116 cells.

**Figure 4:**
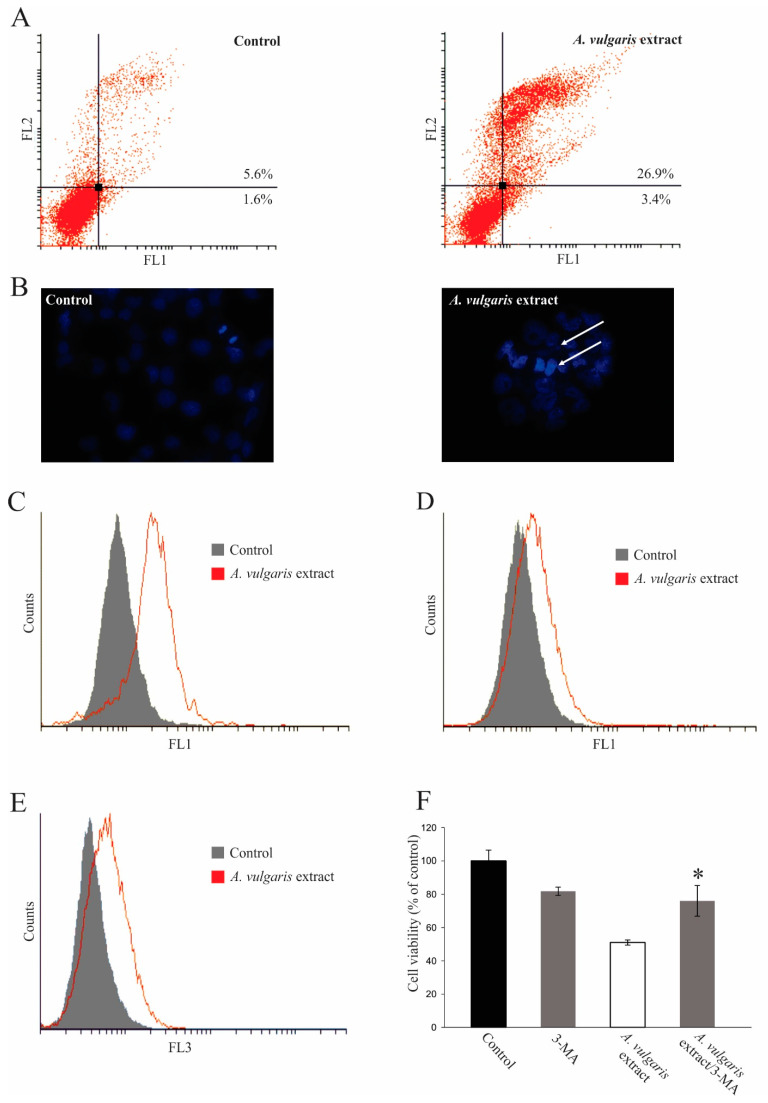
*A. vulgaris* extract displays cytocidal effects as well as inhibition of proliferation of the A549 cell line. Cells were exposed to an IC_50_ dose of *A. vulgaris* extract for 72 h, and flow cytometric analyses of (**A**) Ann/PI staining, (**B**) DAPI staining on chamber slides, (**C**) Apostat staining, (**D**) CFSE staining, and (**E**) AO staining were performed. White arrows mark apoptotic nuclei. (**F**) Cell viability determination after treatment with *A. vulgaris* extract alone and in combination with autophagy inhibitor 3-MA by MTT assay. * *p* < 0.05 was considered statistically significant in comparison to the control.

To define a precise mechanism beyond the effect of *A. vulgaris* extract on cell viability, a flow cytometric analysis of cell death was performed, using the A549 cell line as a representative. Namely, after 72 h of incubation in the presence of an IC_50_ dose of *A. vulgaris* extract, a significant amount of early and late apoptotic cells was detected (Figure 4A). The presence of apoptosis upon the treatment was further confirmed on a morphological level, using DAPI staining of cellular nuclei. Numerous cells with abnormally shaped nuclei and condensed chromatin were visible in cultures exposed to *A. vulgaris* extract (Figure 4B). In concordance with a significant presence of apoptotic cells, amplification of total caspase activity in *A. vulgaris* extract-treated cultures was detected (Figure 4C). On the other hand, the proliferation of survived cells was abrogated, confirming that the extract suppresses cell division as well (Figure 4D). Interestingly, a significant amount of autophagosomes was detected in cells cultivated in the presence of *A. vulgaris* extract (Figure 4E), while the prevention of autophagosome formation in concomitant treatment with inhibitor 3-MA dramatically restored cellular viability (Figure 4F). The obtained result clearly confirmed that the intensified autophagic process triggered by the treatment represented an important part of the cytotoxic activity of the *A. vulgaris* extract.

**Figure 5:**
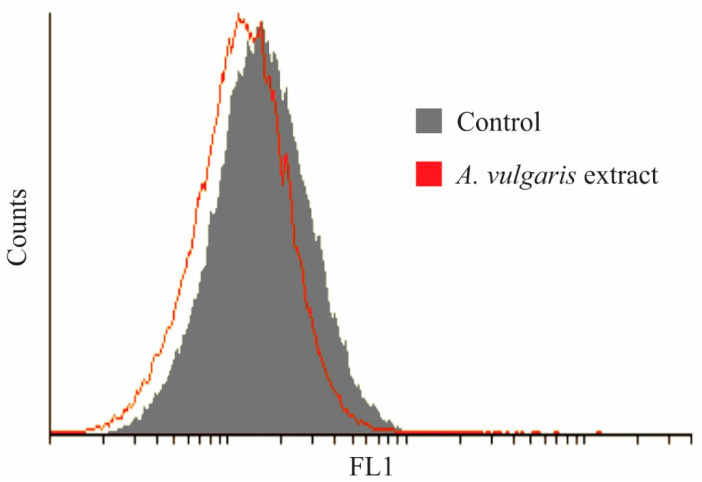
*A. vulgaris* extract slightly inhibits the production of reactive oxygen and nitrogen species. DHR staining of A549 cells exposed to an IC_50_ dose of *A. vulgaris* extract for 72 h.

To exclude the possibility that oxidative stress mediated the antitumor action of *A. vulgaris* extract, the production of hydrogen peroxide and peroxynitrite was estimated by DHR redox-sensitive dye. However, exposure to the *A. vulgaris* extract only slightly inhibited the production of ROS and RNS, indicating their insignificance in triggering the apoptotic process (Figure 5). In summary, the antitumor activity of *A. vulgaris* extract could be ascribed to inhibited proliferation and both caspase-dependent apoptotic and autophagic cell death.

Detailed analysis of the extract content revealed the presence of numerous biologically active compounds with already recognized antitumor potential. Flavonoids belong to a rich group of polyphenolic compounds in the plant kingdom. Numerous data confirm their strong impact on human health, bringing them into the focus of different scientific studies. The members of the flavonol subclass, quercetin, rutin, and isoquercetin, either as glycosides or aglycones, showed antioxidant, antiproliferative, anti-inflammatory, antihypertensive, and antidiabetic effects. The specificity of naturally occurring compounds is in their high adaptability reflected in support of healthy tissues and healing of pathological conditions. This dual potential of certain extracts or separate compounds is often unexplainable, while the mechanisms triggered by them can be even opposite in different tissues and cells dependent on the platform on which they arrived. Therefore, isoquercetin prevented lipid peroxidation through interference with xanthine oxidase activity, chelation of redox-active metals, or direct scavenging of ROS, exhibiting protective features. On the other hand, the same compound affected the signaling pathways involved in tumor progression, such as the Wnt signaling pathway and mitogen-activated protein kinase, directly affecting tumor viability [47]. Similarly, quercetin, apart from its strong cytoprotective abilities, alters cell cycle progression in neoplastic cells, inhibiting their proliferation, inducing programmed cell death types I and II, and blocking metastasis [48]. Importantly, some herbal compounds possess a highly selective potential and act as targeted therapy, affecting certain signaling pathways or molecules important for malignant phenotype maintenance. For example, gallic acid functions as an EGFR antagonist, suppressing EGFR-positive NSCLC progression under certain conditions [49]. In addition, this and similar plant-derived compounds interfered with chemotherapy, enhancing its effectiveness by changing the pro/antiapoptotic molecule ratio [50].

Apart from mentioned quercetin, isoquercetin, and gallic acid, several other constituents that are present in *A. vulgaris* extract might also be beneficial in neoplastic conditions since each of them possesses the potential to directly or indirectly influence disease progression *per se* and in interference with other compounds in the extract. Experience collected from ethnobotanical data and clinical practice confirms that total herbal extracts usually exert more powerful effects than separate compounds. Moreover, some of them, such as quinic acid, have the intrinsic potential to “recognize” selectin-upregulated tumors and induce a transient increase in endothelial permeability to translocate across the endothelial layer. This will result in achieving greater tumor accumulation and delivery of numerous compounds with the direct potential to suppress tumor cell division or viability [51].

The described phenomenon at least partly explains why such compounds can work as destructive for the tumor and protective for normal tissue simultaneously [51]. In this study, the principle of *A. vulgaris* extract duality was illustrated by the genoprotection of primary lymphocytes exposed to a certain dose range of the extract, while the same extract exerts cytotoxic potential against transformed cells in doses 5 to 15 times higher than those applied in the micronucleus assay. Bearing in mind that compounds such as quinic acid with a homing potential for cancer tissue are present in *A. vulgaris* extract, one can expect the multiple beneficial effects of its application *in vivo*.

## 3. Materials and Methods

### 3.1. Plant Material and Extract Preparations

Plant material (official name—*Alchemilla vulgaris* L.; local name—virak; English name—lady’s mantle) was collected in Southeast Serbia, in the region of the Vlasina plateau (N 42.8779987, E 22.0592615) in 2020. The aerial parts were collected at the full flowering phase. Plant material was air-dried and milled before the extraction procedure. Taxonomic and botanical identity was confirmed by Prof. Zora Dajić-Stevanović. The voucher specimen (No. RS-120718-1) is kept in the Herbarium of the Department of Applied Botany, Faculty of Agriculture, University of Belgrade. The species status of *Alchemilla vulgaris* L. is accepted in the relevant plant databases (e.g., http://www.worldfloraonline.org/ (accessed on 21 October 2022)).

After collection, aerial parts of *Alchemilla vulgaris* L. were appropriately air-dried in a well-ventilated room (in shadow at 4 °C) and milled. The amount of 100 g of plant sample was extracted (period of 2 h) in hot ethanol at 60 °C (1 L) three times. After the separation of crude material, collected extracts were combined and evaporated in a vacuum at 60 °C by using a rotary evaporator. The obtained semisolid extract without solvent was sealed and stored at 4 °C for further analysis.

### 3.2. Phytochemical Analyses

The content of selected phytochemicals was determined by application of the standard spectrophotometric methods, namely Folin–Ciocalteu (total phenolics, TPC, New South Wales, Australia), aluminum chloride (total flavonoids, TFC, Daly City, CA, USA) and Arnow’s method (total dihydroxycinnamic acid derivatives, HCA, Nashville, TN, USA), and expressed as mg/g equivalents of gallic acid (GAE), quercetin (QE) and chlorogenic acid (CGAE) calculated on dry weight (DW) of the sample respectively. For deeper phytochemical characterization, UHPLC–HRMS was performed.

#### 3.2.1. UHPLC–HRMS Analysis

UHPLC–HRMS analysis was performed using a Thermo Scientific Dionex Ultimate 3000 RSLC (Germering, Bavaria, Germany) consisting of 6-channel degasser SRD-3600, high-pressure gradient pump HPG-3400RS, autosampler WPS-3000TRS, and column compartment TCC-3000RS coupled to a Thermo Scientific Q Exactive Plus (Bremen, Germany) equipped with heated electrospray ionization (HESI-II). UHPLC separation was achieved on a reversed-phase Kromasil Eternity XT C18 column (Nouryon, Göteborg, Sweden) (2.1 × 100 mm, 1.8 μm) equipped with precolumn SecurityGuard ULTRA UHPLC EVO C18 (Phenomenex, Torrance, CA, USA) maintained at 40 °C. The binary mobile phase consisted of A: 0.1% formic acid in water and B: 0.1% formic acid in acetonitrile. The run time was 34.5 min. The following gradient was used: the mobile phase was held at 5% B for 1 min, gradually turned to 30% B over 24 min, increased gradually to 40% B over 5 min, increased gradually to 95% B over 2.5 min, and held at 95% B for 2 min. The system was then turned to the initial condition of 5% B and equilibrated over 4.5 min. The flow rate and the injection volume were set to 300 µL/min and 2 µL, respectively. The tune parameters of the mass spectrometer were as follows: spray voltage, 2.5 kV; sheath gas flow rate, 38 arbitrary units (a.u.); auxiliary gas flow rate, 12 a.u.; capillary temperature and probe heater temperature, 320 °C; and S-lens RF level, 50. The acquisition was performed in the full-scan MS and data-dependent MS2 modes. Full-scan spectra over the *m*/*z* range of 100 to 1500 were acquired in negative ionization mode at a resolution of 70,000. Other instrument parameters for full MS mode were set as follows: automatic gain control (AGC) target, 3 × 10^6^; maximum injection time (IT), 80 ms. For the ddMS2 mode, the instrument parameters were as follows: resolution, 17,500; AGC target, 1 × 10^5^; maximum IT, 50 ms; Top5; isolation window, 2.0 *m*/*z*; stepped normalized collision energy (NCE), 20, 40, and 60 eV. Data acquisition and processing were carried out with Xcalibur 4.0 software (Thermo Scientific, Inc. Waltham, MA, USA).

#### 3.2.2. Total Phenolic Content (TPC)

The Folin–Ciocalteu (FC) method was used for TPC determination according to [52]. Results were expressed as milligrams of ferulic acid equivalents (FAEs) per gram of dry weight (DW).

#### 3.2.3. Total Flavonoid Content (TFC)

The determination of TFC was evaluated using the spectrophotometric method as described in [53]. TFC was determined using a calibration curve with quercetin (Q) as a standard, and the results were expressed as milligrams of quercetin equivalents (QEs) per gram of DW.

#### 3.2.4. Total Dihydroxycinnamic Acid Derivative Content (HCA)

Total HCA content was estimated using the method described in [54]. The total HCA content in the extract was determined from the calibration curve with chlorogenic acid (CGA) as a standard. Results were expressed as milligrams of CGA equivalents (CGAEs) per gram of DW.

### 3.3. Antioxidant Activity Assays

#### 3.3.1. DPPH (2,2′-Diphenyl-1-picrylhydrazyl Radical) (DPPH) Assay

The determination of the free radical scavenging activity of the extracts was performed according to the method described in [55]. The percentage inhibition of DPPH was calculated by using the following formula:

% of inhibition = [A_b_ − A_s_]/A_b_ × 100 (1)

A_b_—the absorbance of blank; A_s_—the absorbance of the sample extract.

#### 3.3.2. Ferric Reducing Power (FRP) Assay

The antioxidant activity of the *A. vulgaris* extract was determined by FRP assay according to the method previously described in [56]. Ascorbic acid (AA) was used as standard, and obtained results were expressed as milligrams of ascorbic acid equivalents (AAEs) per gram of DW.

#### 3.3.3. Cupric Reducing Antioxidant Activity (CUPRAC) Assay

The CUPRAC assay was performed according to the procedure described in [57]. A calibration curve was prepared using different concentrations of ascorbic acid as a standard, and results were expressed as milligrams of ascorbic acid equivalents (AAEs) per gram of DW.

#### 3.3.4. Total Antioxidant Capacity (TAC) Assay

The TAC assay was conducted using the method given in [58]. The antioxidant capacity was calculated according to a calibration curve prepared with ascorbic acid as standard. The results were expressed as milligrams of ascorbic acid equivalents (AAEs) per gram of DW.

In each method, all samples were analyzed in triplicates (*n* = 3). The absorbance of the resulting solution was measured with a UV/visible spectrophotometer.

### 3.4. In Vitro Antitumor Study

#### 3.4.1. Reagents and Cells

RPMI-1640 medium and fetal bovine serum (FBS) were obtained from Capricorn Scientific GmbH (Hessen, Germany). Phosphate-buffered saline (PBS), dimethyl sulfoxide (DMSO), acridine orange (AO), propidium iodide (PI), sulforhodamine B (SRB), carboxyfluorescein diacetate succinimidyl ester (CFSE), and 3-methyl adenine (3-MA) were purchased from Sigma (St. Louis, MO, USA). The penicillin–streptomycin solution was bought from Biological Industries (Cromwell, CT, USA). Annexin V-FITC (AnnV) was acquired from BD (Pharmingen, San Diego, SAD). DAPI fluoromounth G was bought from Southern Biotech (Birmingham, AL, USA). Dihydrorhodamine 123 (DHR) was from Thermo Fisher Scientific (Waltham, MA, USA). 3-(4,5 dimethythiazol-2-yl)-2,5-diphenyltetrazolium bromide (MTT) was obtained from AppliChem (Darmstadt, Germany), while ApoStat was from R&D Systems (Minneapolis, MN, USA). A375 (human melanoma), HCT116 (human colorectal carcinoma), A549 (human lung carcinoma), and MCF-7 (human breast adenocarcinoma) cell lines were purchased from American Type Culture Collection (ATCC, Rockville, MD, USA).

All cells were routinely maintained in HEPES-buffered RPMI-1640 medium supplemented with 10% heat-inactivated FBS, 2 mM L-glutamine, 0.01% sodium pyruvate, 100 U/mL penicillin, and 100 μg/mL streptomycin. Cells were grown at 37 °C in a humidified atmosphere with 5% CO_2_. The density of MCF-7, HCT116, and A375 at seeding time in 96-well plates for determination of cell viability was 4 × 10^3^ cells/well, and that of A549 was 2 × 10^3^ cells/well. For flow cytometric analyses concerning the A549 cell line in 6-well plates, the density was 7 × 10^4^ cells/well.

Peritoneal exudate cells were collected by lavage with ice-cold PBS from the peritoneal cavity of C57BL/6 mice. Mice originated from our own animal facility at the Institute for Biological Research “Siniša Stanković” (IBISS)—National Institute of the Republic of Serbia, University of Belgrade (Belgrade, Serbia). Exudate cells were cultivated in HEPES-buffered RPMI-1640 medium supplemented with 5% heat-inactivated FBS, 2 mM L-glutamine, 0.01% sodium pyruvate, penicillin (100 units/mL), and streptomycin (100 μg/mL) at 37 °C in a humidified atmosphere with 5% CO_2_. Afterward, cells were counted, seeded in 96-well plates at a density of 1.5 × 10^5^ cells/well, and left for 2 h to adhere. Prior to treatment, non-adherent cells were removed. The handling of animals and the study protocol were in agreement with the local guidelines and the European Community guidelines (EEC Directive of 1986; 86/609/EEC) and approved by the local Institutional Animal Care and Use Committee (IACUC). The approval for the experimental protocols (permission No. 323-07-120098/2020-05) was granted from the national licensing committee at the Department of Animal Welfare, Veterinary Directorate, Ministry of Agriculture, Forestry and Water Management of the Republic of Serbia.

*Alchemilla vulgaris* ethanolic extract stock solution was prepared in DMSO at a concentration of 200 mg/mL before the usage, and the final concentration of DMSO in working solutions was 1%.

#### 3.4.2. Determination of Cell Viability by SRB and MTT Assays

All cell lines were seeded overnight and exposed to a wide range of concentrations of *A. vulgaris* extract for 72 h; after the incubation period, cell viability was assessed using SRB and MTT assays.

For detection of mitochondrial dehydrogenase activity, cells were incubated with MTT solution (0.5 mg/mL) for approximately half an hour until purple formazan crystals were formed. Afterward, the dye was discarded and DMSO was added to dissolve formazan. For the SRB assay, cells were fixed with 10% TCA for 2 h at 4 °C and stained with 0.4% SRB solution for 30 min at RT. Stained cells were dissolved in 1% acetic acid, washed, and dried overnight. The absorbance of dissolved dye (in 10 mM TRIS buffer for 20 min) was measured at 540 nm. Cell viability was calculated as a percentage of control that was arbitrarily set to 100%.

#### 3.4.3. AnnV/PI, Apostat, and AO Staining

For all flow cytometric analyses, A549 cells were seeded overnight and treated with an IC50 dose of A. vulgaris extract (35 μg/mL) for 72 h.

For caspase activation detection, cells were stained with pan-caspase inhibitor Apostat in accordance with the manufacturer’s protocol. For the detection of autophagosomes, cells were stained with a solution of 1 µg/mL AO for 15 min at 37 °C. For the detection of apoptotic cell death, cells were stained with 15 µg/mL Annexin V-FITC and 15 µg/mL PI and analyzed using CyFlow Space Partec using the PartecFloMax software (Munster, Germany).

#### 3.4.4. CFSE Staining

Prior to treatment, A549 cells were stained with CFSE to a final concentration of 1 μM and incubated for 10 min at 37 °C. Afterward, cells were washed, seeded overnight, and treated with an IC_50_ dose of *A. vulgaris* extract (35 μg/mL) for 72 h and then analyzed using flow cytometry.

#### 3.4.5. Measurement of ROS/RNS Generation

By measuring the intensity of green fluorescence emitted by redox-sensitive dye DHR, the production of reactive oxygen and nitrogen species was detected. A549 cells were incubated with DHR for 20 min at 37 °C, seeded overnight, and treated with an IC_50_ dose of *A. vulgaris* extract (35 μg/mL) for 72 h. At the end of the incubation period, cells were analyzed by flow cytometry.

#### 3.4.6. DAPI Staining on Chamber Slides

To evaluate morphological signs of apoptosis, A549 cells were seeded in 4-chamber slides at 1 × 10^4^ cells/well density and treated with an IC_50_ dose (35 μg/mL) of *A. vulgaris* extract for 72 h. Afterward, cells were fixed with 4% (*w/v*) paraformaldehyde for 15 min at RT, washed, and covered with DAPI fluoromounth-G before analysis. Chamber slides were analyzed using Zeiss AxioObserver Z1 inverted fluorescence microscope (Carl Zeiss AG, Oberkochen, Germany) at 400× magnification.

### 3.5. Cytokinesis-Blocked Micronucleus (CBMN) Assay

Venous blood samples were obtained with heparinized sterile vacutainers from 4 healthy female volunteers (2 × 5 mL from each) who had not been exposed to chemicals, drugs, or other substances. The volunteers signed informed consent and gave permission for the use of their blood for experimental purposes. The study complied with the code of ethics of the World Medical Association (Helsinki Declaration of 1975, as revised in 2013). The blood samples were obtained at the medical unit of the Nuclear Facilities of Serbia, the Institute of Nuclear Sciences “Vinca”, in accordance with current (2005) Serbian health and ethical regulations.

Human peripheral blood lymphocyte (2 × 10^6^) cultures resuspended in 5 mL of RPMI-1640 medium supplemented with 15% calf serum and 2.4 μg/mL of phytohemagglutinin (PHA), (Invitrogen-Gibco-BRL) were treated with three different concentrations of *A. vulgaris* extract (2 μg/mL, 4 μg/mL, and 6 μg/mL) after 1 h exposure to PHA. A cell culture containing Amifostine WR-2721 (Marligen-Biosciences, MD, USA) (1 µg/mL) served as positive control, while a culture with mitomycin C (MMC) (0.2 μg/mL, in phosphate buffer) was the negative control. All cell cultures were incubated at 37 °C. Treatment with *A. vulgaris* extract lasted for 19 h, after which cell cultures were rinsed with pure medium, transferred into 5 mL fresh RPMI 1640 medium (RPMI 1640 Medium + GlutaMAX + 25 mM HEPES; Invitrogen-Gibco-BRL) and incubated for an additional 72 h. The incidence of spontaneously occurring micronuclei (MNs) in control samples was scored.

For the preparation of MNs, the modified cytokinesis block method [59,60] was used. Cytochalasin B (Invitrogen-Gibco-BRL) was added to samples after 44 h of culture at a final concentration of 6 μg/mL, and the lymphocyte cultures were incubated for another 24 h. After 72 h, cells were washed with 0.9% NaCl (Merck, Sharp, & Dohme GMBH, Wien, Austria), collected by centrifugation, and treated with the hypotonic solution at 37 °C. The hypotonic solution consisted of 0.56% KCl + 0.9% NaCl (mixed in equal volumes). The cell suspension was prefixed in methanol/acetic acid (3:1), washed three times with fixative, and dropped onto a clean slide [59]. The slides were air-dried and stained with alkaline Giemsa (Sigma-Aldrich, St. Louis, MO, USA) (2%). At least 1000 binucleated (BN) cells per sample were scored, and MN was registered according to the criteria of [59,60].

Since micronucleus expression is dependent on cell proliferation, quantification of cell proliferation and cell death should be carried out to obtain a sound evaluation of cell kinetics and micronucleus frequencies. The CBPI was calculated as suggested in [59].

The number of binucleated cells with one, two, three, or more MNs were then tabulated. The data for each treatment were expressed as the frequency of MNs per 1000 binucleated cells.

### 3.6. Statistical Analysis

Student’s *t*-test was used to evaluate the significance of differences between groups, and *p*-values of less than 0.05 were considered to indicate statistical significance.

For the micronucleus assay, the statistical significance of the difference between the data pairs was evaluated by analysis of variance (one-way ANOVA) followed by the Tukey test. Statistical difference was considered significant at *p* < 0.01. The calculated index is presented as the percentage of change between different groups.

## 4. Conclusions

The results of this study strongly support the historically collected data about the healing potential of *Alchemilla vulgaris* L. from Southeast Europe, which was traditionally used as a medicinal plant for centuries. This study confirmed that the ethanolic extract of *Alchemilla vulgaris* L. represents a valuable source of bioactive compounds with multiple beneficial biological properties, including strong antitumor activity and remarkable genoprotective features resulting, at least partly, from the strong antioxidant potential of this plant. Further research on the antitumor activity of lady’s mantle should target the effects of individual components of its extract, as well as the effects of possible synergistic activity of different bioactive compounds, in addition to revealing their complex mechanisms responsible for anticancer action. All of the findings mentioned above make this plant a valuable candidate for further research in the field of drug discovery.

## Figures and Tables

**Table 1:** Compounds detected and tentatively characterized in *A. vulgaris* extract by UHPLC-HRMS.

No.	RT (min)	Tentative Identification	Ion Species	Precursor Ion (*m*/*z*)	δ ppm	Molecular Formula	Product Ions (*m*/*z*)	Reference
1	1.68	Gallic acid	[M − H]^−^	169.01331	1.66	C_7_H_5_O_5_	169, **125**	[17]
2	6.59	*p*-Coumaroyl hexose 1	[M − H]^−^	325.09314	4.14	C_15_H_17_O_8_	325, 163, **145**, 119, 117	[18]
3	6.69	Chlorogenic acid	[M − H]^−^	353.08835	4.64	C_16_H_17_O_9_	**191**, 173, 179, 135	[18]
4	6.73	Galloyl-HHDP-hexose	[M − H]^−^	633.07430	1.52	C_27_H_21_O_18_	633, 463, **301**, 275, 229	[19]
5	6.98	Brevifolin carboxylic acid	[M − H]^−^	291.01489	4.64	C_13_H_7_O_8_	291, **247**, 219, 191	[20]
6	7.08	*p*-Coumaroyl hexose 2	[M − H]^−^	325.09314	4.54	C_15_H_17_O_8_	325, 163, **145**, 119, 117	[18]
7	7.14	Caffeic acid	[M − H]^−^	179.03413	1.39	C_9_H_7_O_4_	179, **135**, 91	[17]
8	8.06	Quercetin-hexoside-glucuronide	[M − H]^−^	639.12152	1.93	C_27_H_27_O_18_	639, 463, **301**, 151	[19]
9	8.62	Quercetin-3-*O*-arabinoside-7-*O*-glucoside	[M − H]^−^	595.13129	1.40	C_26_H_27_O_16_	595, 463, 462, 433, 301, **299**, 271	[21]
10	9.10	*p*-Coumaroylquinic acid 1	[M − H]^−^	337.09344	4.90	C_16_H_17_O_8_	337, **191**, 163, 145, 119	[18]
11	9.78	Syringic acid	[M − H]^−^	197.04489	2.24	C_9_H_9_O_5_	**197**, 169, 153, 125	[22]
12	9.93	Myricetin 3-*O*-hexoside (glucoside or galactoside)	[M − H]^−^	479.08360	1.82	C_21_H_19_O_13_	**479**, 317, 316, 287, 271, 179, 165, 139	[21]
13	10.12	*p*-Coumaric acid	[M − H]^−^	163.03917	1.22	C_9_H_7_O_3_	163, 119	[17]
14	10.24	Brevifolin	[M − H]^−^	247.02469	3.94	C_12_H_7_O_6_	**247**, 219, 191, 173	[20]
15	10.42	*p*-Coumaroylquinic acid 2	[M − H]^−^	337.09335	4.63	C_16_H_17_O_8_	337, **191**, 163, 119	[18]
16	10.43	Ellagic acid hexose	[M − H]^−^	463.05243	3.70	C_20_H_15_O_13_	463, **301**, 283, 229	[23]
17	10.46	Gossypetin-7-*O*-rhamnoside-3-*O*-hexoside	[M − H]^−^	625.14417	5.03	C_27_H_29_O_17_	625, **479**, 463, 317, 316, 315, 287, 271	[24]
18	10.63	Gossypetin-7-*O*-rhamnoside-3-*O*-deoxyhexoside 1	[M − H]^−^	595.13116	1.18	C_26_H_27_O_16_	595, 463, 462, **449**, 317, 316, 315, 287, 271	[24]
19	10.65	Gossypetin-7-*O*-rhamnoside-3-*O*-deoxyhexoside 2	[M − H]^−^	595.13116	1.18	C_26_H_27_O_16_	595, 463, 462, **449**, 317, 316, 315, 287, 271	[24]
20	12.59	Quercetin-3-*O*-vicianoside (Quercetin 3-*O*-α-L-arabinopyranosyl-(1–6)-β-D-glucopyranoside)	[M − H]^−^	595.12927	−1.99	C_26_H_27_O_16_	595, 301, **300**, 271, 255, 179	[21]
21	12.86	Quercetin-3-*O*-pentoside–7-*O*-deoxyhexoside	[M − H]^−^	579.13635	3.29	C_26_H_27_O_15_	579, 447, 446, 433, 301, **299**, 271, 179, 151	[21]
22	12.96	Ellagic acid pentose	[M − H]^−^	433.04150	3.12	C_19_H_13_O_12_	433, **301**, 283, 229, 185	[23]
23	13.61	Ellagic acid	[M − H]^−^	300.99902	0.10	C_14_H_5_O_8_	**301**, 283, 245, 229, 201, 185, 173, 145	[23]
24	13.82	Rutin	[M − H]^−^	609.1473	1.96	C_27_H_30_O_16_	609, **300**, 271, 255, 179, 151	[18]
25	13.94	Hyperoside	[M − H]^−^	463.08862	3.28	C_21_H_19_O_12_	463, **300**, 301, 271, 255, 179, 151	[18]
26	14.02	Miquelianin (Quercetin 3-*O*-glucuronide)	[M − H]^−^	477.06735	2.07	C_21_H_17_O_13_	477, **301**, 255, 179, 151	[19]
27	14.10	Agrimoniin	[M − 2H]^−2^	934.0721	0.78	C_82_H_54_O_52_	1567, 1265, 1085, 935, 897, 783, 633, **301**	[19]
28	14.25	Kaempferol-3-*O*-rutinoside	[M − H]^−^	593.15198	3.17	C_27_H_29_O_15_	593, **285**	[22]
29	14.28	Isoquercitrin (Quercetin 3-*O*-glucoside)	[M − H]^−^	463.08875	3.55	C_21_H_19_O_12_	463, 301, **300**, 271, 255, 179, 151	[18]
30	14.44	Cynaroside (luteolin 7-*O*-glucoside)	[M − H]^−^	447.09378	3.56	C_21_H_19_O_11_	447, **285**, 199	[22]
31	15.05	Guaiaverin (quercetin 3-*O*-α-L-arabinopyranoside)	[M − H]^−^	433.07797	3.31	C_20_H_17_O_11_	433, **300**, 301, 271, 255, 179, 151	[18]
32	15.44	Avicularin (quercetin 3-*O*-α-L-arabinofuranoside)	[M − H]^−^	433.07773	2.75	C_20_H_17_O_11_	433, **300**, 301, 271, 255, 179, 151	[18]
33	16.16	Kaempferol 3-*O*-glucuronide	[M − H]^−^	461.07315	3.68	C_21_H_17_O_12_	461, **285**, 229	[19]
34	16.29	Astragalin (Kaempferol 3-*O*-glucoside)	[M − H]^−^	447.09375	3.49	C_21_H_19_O_11_	**447**, 300, 285, 284, 255, 227	[18]
35	17.04	Kaempferol 3-*O*-xyloside	[M − H]^−^	417.08323	3.83	C_20_H_17_O_10_	**417**, 285, 284, 255, 227	[22]
36	18.83	Quercetin 3-*O*-(6-*O*-acetyl-β-D-glucopyranoside	[M − H]^−^	505.09930	3.24	C_23_H_21_O_13_	505, 301, **300**, 271, 255, 179, 151	[22]
37	20.78	Triterpene acid hexoside	[M + HCOO]^−^	711.39728	1.64	C_37_H_59_O_13_	**503**	[25]
38	21.09	Chrysoeriol 7-*O*-glucuronide	[M − H]^−^	475.08850	2.94	C_22_H_19_O_12_	**299**, 284, 255	[26]
39	21.31	Kaempferol 3-*O*-acetylglucoside	[M − H]^−^	489.10428	3.12	C_23_H_21_O_12_	489, 284, **255**, 227	[27]
40	21.45	Quercetin	[M − H]^−^	301.03546	3.95	C_15_H_9_O_7_	**301**, 179, 151	[28]
41	23.21	Tiliroside (kaempferol 3-*O*-(6′′-*O*-p-coumaroyl)-β-D-glucopyranoside)	[M − H]^−^	593.13086	3.19	C_30_H_25_O_13_	593, **285**, 255, 227	[25]
42	27.18	Triterpene acid hexoside	[M + HCOO]^−^	695.40216	1.38	C_37_H_59_O_12_	**487**	[25]
43	27.78	Triterpene acid hexoside	[M + HCOO]^−^	695.40216	1.38	C_37_H_59_O_12_	**487**	[25]
44	27.87	Triterpene acid hexoside	[M + HCOO]^−^	695.40216	1.38	C_37_H_59_O_12_	**487**	[25]
45	29.83	Arjungenin	[M − H]^−^	503.33853	3.61	C_30_H_47_O_6_	**503**, 441, 409	[29]

The values in bold correspond to the base peak.

**Table 2:** Total phenolics and flavonoids and antioxidant activity.

	TPC mg/g GAE	TFC mg/g QE	HCA mg/g CGAE	CUPRAC mg/g AAE	TAC mg/g GAE	DPPH μg/gsr Trolox	FRP mg/g GAE
mean	7.55	6.99	14.18	2.61	326.50	18.02	31.32
SD	0.43	0.14	0.28	0.09	1.12	0.02	0.26

**Table 3:** Incidence of MN, cytokinesis-block proliferation index, distribution of MN per cell and frequency of MN, measurement in cell cultures of human lymphocytes treated with different concentrations of *A. vulgaris* extract.

Conc. µg/mL	% Bn Cell with MN	MN/Bn Cell	CBPI	Frequency of MN
Control	2.1 ± 0.11	1.2 ± 0.06	1.6 ± 0.02	100%
Amifos.—1.0 µg/mL	1.8 ± 0.14	1.2 ± 0.09	1.7 ± 0.02	(81.4%) − 18.6%
MMC—0.2 µg/mL	2.8 ± 0.10	1.2 ± 0.02	1.6 ± 0.03	(124.2%) + 24.2%
*A. vulgaris*—2 µg/mL	1.7 ± 0.09	1.2 ± 0.06	1.7 ± 0.01	(79.5%) − 20.5%
*A. vulgaris*—4 µg/mL	1.6 ± 0.03	1.3 ± 0.05	1.7 ± 0.06	(81.8%) − 18.2%
*A. vulgaris*—6 µg/mL	1.8 ± 0.06	1.2 ± 0.04	1.6 ± 0.02	(83.7%) − 16.3%

% Bn cells with micronuclei. MN/Bn cells—incidence of micronuclei in binucleated cells. CBPI—cytokinesis-block proliferation index. Frequency of MN—incidence of MN present as % from control groups in cell cultures of human lymphocytes treated with different concentrations of *A. vulgaris* extract.

**Table 4:** IC_50_ values of *A. vulgaris* extract-treated cell lines determined after 72 h.

Assays	MCF-7 µg/mL	A375 µg/mL	A549 µg/mL	HCT116 µg/mL
MTT	83.5 ± 8.9 *	105.8 ± 8.7	36.1 ± 5.7	36.9 ± 5.6
SRB	80.3 ± 0.4	106.4 ± 8.9	30.3 ± 8.3	54.9 ± 1.4

* Average ± SD.

## Data Availability

Data supporting obtained results can be obtained from the authors upon request.

## Sample Availability

Samples of the *A. vulgaris* extract are not available from the authors.
